# Baseline Chest Computed Tomography as Standard of Care in High-Risk Hematology Patients

**DOI:** 10.3390/jof6010036

**Published:** 2020-03-13

**Authors:** Jannik Stemler, Caroline Bruns, Sibylle C. Mellinghoff, Nael Alakel, Hamdi Akan, Michelle Ananda-Rajah, Jutta Auberger, Peter Bojko, Pranatharthi H. Chandrasekar, Methee Chayakulkeeree, José A. Cozzi, Elizabeth A. de Kort, Andreas H. Groll, Christopher H. Heath, Larissa Henze, Marcos Hernandez Jimenez, Souha S. Kanj, Nina Khanna, Michael Koldehoff, Dong-Gun Lee, Alina Mager, Francesco Marchesi, Rodrigo Martino-Bufarull, Marcio Nucci, Jarmo Oksi, Livio Pagano, Bob Phillips, Juergen Prattes, Athina Pyrpasopoulou, Werner Rabitsch, Enrico Schalk, Martin Schmidt-Hieber, Neeraj Sidharthan, Pere Soler-Palacín, Anat Stern, Barbora Weinbergerová, Aline El Zakhem, Oliver A. Cornely, Philipp Koehler

**Affiliations:** 1 Department I of Internal Medicine, Center for Integrated Oncology Aachen Bonn Cologne Duesseldorf (CIO ABCD), Excellence Center for Medical Mycology (ECMM), University of Cologne, Faculty of Medicine and University Hospital Cologne, 50937 Cologne, Germany; caroline.bruns@uk-koeln.de (C.B.); sibylle.mellinghoff@uk-koeln.de (S.C.M.); oliver.cornely@uk-koeln.de (O.A.C.); philipp.koehler@uk-koeln.de (P.K.); 2 Cologne Excellence Cluster on Cellular Stress Responses in Aging-Associated Diseases (CECAD), University of Cologne, 50931 Cologne, Germany; 3 German Centre for Infection Research, Partner Site Bonn-Cologne, 50937 Cologne, Germany; 4 Department of Internal Medicine I, University Hospital of Dresden, 01307 Dresden, Germany; nael.alakel@uniklinikum-dresden.de; 5 Hematology Clinical Research Unit, Cebeci Hospital, Ankara University Faculty of Medicine, 06100 Ankara, Turkey; hamdiakan@gmail.com; 6 Dept of Infectious Diseases and General Medical Unit, Alfred Health & Central Clinical School, Monash University, Melbourne 3004, Australia; michelle.ananda-rajah@monash.edu; 7 Onkologische Schwerpunktpraxis Freilassing, 83395 Freilassing, Germany; auberger@onko-endo.de; 8 Department of Hematology and Oncology, Red Cross Hospital Munich, 80634 Munich, Germany; peter.bojko@swmbrk.de; 9 Division of Infectious Diseases, Wayne State University School of Medicine, Karmanos Cancer Center, Detroit, MI 48201, USA; pchandrasekar@med.wayne.edu; 10 Division of Infectious Diseases and Tropical Medicine, Department of Medicine, Faculty of Medicine Siriraj Hospital, Mahidol University, Bangkok 10700, Thailand; methee.cha@mahidol.ac.th; 11 Hematology Department, Hospital Provincial Del Centenario, Rosario 2000, Argentina; joseacozzi@hotmail.com; 12 Department of Hematology, Radboud University Medical Center, 6500 Nijmegen, The Netherlands; Elizabeth.Dekort@Radboudumc.nl; 13 Infectious Disease Research Program, Center for Bone Marrow Transplantation and, Department of Pediatric Hematology and Oncology, University Children’s Hospital, 48149 Münster, Germany; grollan@ukmuenster.de; 14 Department of Microbiology (PathWest Laboratory Medicine, WA, FSH Network), Perth 6000, Australia; chris.heath@health.wa.gov.au; 15 Depts. of Infectious Diseases, Fiona Stanley Hospital & Royal Perth Hospital, Perth 6000, Australia; 16 Faculty of Health & Medical Sciences, University of Western Australia, Murdoch/Perth, Murdoch 6150, Australia; 17 Department of Medicine, Clinic III – Hematology, Oncology, Palliative Medicine, Rostock University Medical Center, 18057 Rostock, Germany; larissa.henze@med.uni-rostock.de; 18 Head of the bone marrow unit, Hospital City Dr. Enrique Tejera, 2001 Valencia, Venezuela; mhernanj@gmail.com; 19 Departament of Medicine, Facultad de Ciencias de la Salud, University of Carabobo, 2001 Valencia, Venezuela; 20 Division of Infectious Diseases, Infection Control Program, Antimicrobial Stewardship Program, American University of Beirut Medical Center, Beirut 1107 2020, Lebanon; sk11@aub.edu.lb; 21 Division of Infection Diseases and Hospital Epidemiology, University and University Hospital of Basel, 4031 Basel, Switzerland; nina.khanna@usb.ch; 22 Department of Bone Marrow Transplantation, West German Cancer Center, University Hospital Essen, University of Duisburg-Essen, 45147 Essen, Germany; michael.koldehoff@uk-essen.de; 23 Division of infectious Diseases, Department of Internal Medicine, Catholic Hematology Hospital & Seoul St. Mary’s Hospital, College of Medicine, The Catholic University of Korea, 06591 Seoul, Korea; symonlee@catholic.ac.kr; 24 Department of Diagnostic and Interventional Radiology, University of Cologne, Faculty of Medicine and University Hospital Cologne, 50937 Cologne, Germany; Alina.mager@uk-koeln.de; 25 Hematology and Stem Cell Transplant Unit, IRCCS Regina Elena National Cancer Institute, Via Elio Chianesi, 53 00144 Rome, Italy; francesco.marchesi@ifo.gov.it; 26 Servei d’Hematologia Clinica, Hospital de la Santa Creu i Sant Pau, 08041 Barcelona, Spain; rmartino@santpau.cat; 27 Department of Internal Medicine, Universidade Federal do Rio de Janeiro, Rio de Janeiro 21941-901, Brazil; mnucci@hucff.ufrj.br; 28 Department of Infectious Diseases, Turku University Hospital and University of Turku, 20521 Turku, Finland; Jarmo.Oksi@tyks.fi; 29 Dipartimento di Diagnostica per Immagini, Radioterapia Oncologica ed Ematologia, Fondazione Policlinico A. Gemelli -IRCCS, 00169 Rome, Italy; Livio.Pagano@unicatt.it; 30 Sezione di Ematologia, Dipartimento di Scienze Radiologiche ed Ematologiche, Università Cattolica del Sacro Cuore, 00168 Rome, Italy; 31 Leeds Children’s Hospital, Leeds General Infirmary, Leeds Teaching Hospitals, NHS Trust, Leeds LS1 3EX, UK; bob.phillips@york.ac.uk; 32 Centre for Reviews and Dissemination, Alcuin College, University of York, York YO10 5DD, UK; 33 Department of Internal Medicine, Section of Infectious Diseases and Tropical Medicine, Medical University of Graz, 8036 Graz, Austria; juergen.prattes@medunigraz.at; 34 Infectious Diseases Unit, Hippokration Hospital, 54642 Thessaloniki, Greece; a.pyrpasopoulou@doctors.org.uk; 35 Department of Internal Medicine I, Bone Marrow Transplant-Unit, Medical University of Vienna, 1090 Vienna, Austria; werner.rabitsch@meduniwien.ac.at; 36 Department of Hematology and Oncology, Otto-von-Guericke University Magdeburg, Medical Center, 39120 Magdeburg, Germany; enrico.schalk@med.ovgu.de; 37 2. Medizinische Klinik, Carl-Thiem-Klinikum Cottbus, 03048 Cottbus, Germany; m.schmidt_hieber@ctk.de; 38 Department of Clinical Haematology, Amrita Institute of Medical Sciences, Kochi 682041, India; neerajsidh@gmail.com; 39 Pediatric Infectious Diseases and Immunodeficiencies Unit. Vall d’Hebron Barcelona Hospital Campus, 08035 Barcelona, Spain; psoler@vhebron.net; 40 Infectious Diseases institute, Rambam Health Care Campus, 3109601 Haifa, Israel; a_shteren@rambam.health.gov.il; 41 Department of Internal Medicine–Hematology and Oncology, Masaryk University and University Hospital Brno, 62500 Brno, Czech Republic; weinbergerova.barbora@fnbrno.cz; 42 Division of Infectious Diseases, American University of Beirut Medical Center, Beirut 1107 2020, Lebanon; az51@aub.edu.lb; 43 Clinical Trials Centre Cologne, ZKS Köln, 50935 Cologne, Germany

**Keywords:** invasive aspergillosis, antifungal prophylaxis, infection in hematology

## Abstract

Baseline chest computed tomography (BCT) in high-risk hematology patients allows for the early diagnosis of invasive pulmonary aspergillosis (IPA). The distribution of BCT implementation in hematology departments and impact on outcome is unknown. A web-based questionnaire was designed. International scientific bodies were invited. The estimated numbers of annually treated hematology patients, chest imaging timepoints and techniques, IPA rates, and follow-up imaging were assessed. In total, 142 physicians from 43 countries participated. The specialties included infectious diseases (*n* = 69; 49%), hematology (*n* = 68; 48%), and others (*n* = 41; 29%). BCT was performed in 57% (*n* = 54) of 92 hospitals. Upon the diagnosis of malignancy or admission, 48% and 24% performed BCT, respectively, and X-ray was performed in 48% and 69%, respectively. BCT was more often used in hematopoietic cell transplantation and in relapsed acute leukemia. European centers performed BCT in 59% and non-European centers in 53%. Median estimated IPA rate was 8% and did not differ between BCT (9%; IQR 5–15%) and non-BCT centers (7%; IQR 5–10%) (p = 0.69). Follow-up computed tomography (CT) for IPA was performed in 98% (*n* = 90) of centers. In high-risk hematology patients, baseline CT is becoming a standard-of-care. Chest X-ray, while inferior, is still widely used. Randomized, controlled trials are needed to investigate the impact of BCT on patient outcome.

## 1. Introduction

Invasive aspergillosis (IA) typically affects high-risk hematology patients, in particular those with acute leukemia or the recipients of hematopoietic cell transplantation (HCT) [1]. The incidence of probable or proven IA in these patients ranges from 2%, while on posaconazole prophylaxis to 11.2% without mold-directed prophylaxis [2,3]. The overall and attributable mortality are high reaching up to 42% and 27%, respectively [4,5]. Invasive pulmonary aspergillosis (IPA) is associated with even higher mortality up to 75%, and it has been shown to negatively impact the long-term survival of leukemia patients [6,7].

Early diagnosis and treatment are crucial in the management of IPA to improve patient outcome [8,9]. However, IA is still a frequently missed diagnosis in hematological patients [10]. In patients that were diagnosed with IPA, the serial assessment of pulmonary findings on chest CT has been shown to be superior when compared to galactomannan or lesion counts in survival prediction and combination of these tools seems useful [11].

Baseline chest computed tomography (BCT) in adult high-risk hematology patients has been suggested for the early diagnosis of IPA. Abnormal findings on BCT were found to be an independent risk factor for invasive fungal disease (IFD) [12]. Recent prospective studies in patients that were admitted for intensive chemotherapy or HCT found abnormalities on BCT in 36% (*n* = 196) of patients close to admission time, and 10% met the EORTC/MSG radiographic consensus criteria for IFD [13]. When BCT findings were abnormal, the risk of developing IPA doubled as compared to unremarkable BCT findings [14]. A study that was conducted in Israel found abnormal BCT in 31% (*n* = 295) of patients; of these, 5% were diagnosed with IPA on admission and another 10% subsequently during hospital stay. In the subgroup of patients with de novo acute myeloid leukemia (AML), 55% of IPA were detected by BCT [15]. However, none of this has been the subject of randomized, controlled trials, yet complicating the impact assessment of BCT on mortality and follow-up.

BCT appears to be a useful screening tool for identifying those patients that require treatment for IPA rather than prophylaxis. However, the added value might depend on local epidemiology, exact timing of the CT, specific imaging techniques, and it might differ between patient groups. Since this patient population frequently receives mold-active prophylaxis during remission induction chemotherapy, BCT might help to differentiate primary IPA from breakthrough invasive fungal infection (BT-IFI) [16]. The guidelines do not recommend BCT in this high-risk group [9,17]. Currently, it is unknown how widely BCT has been implemented and what the specifics of BCT use in hematology departments are. Therefore, we conducted a survey to determine the current BCT practice in hematology departments throughout the world.

## 2. Materials and Methods

A web-based health services research questionnaire was designed and made accessible via www.clinicalsurveys.net.

Members of the following scientific bodies were invited to participate: Working group on Infections in Hematology and Oncology of the German Society for Hematology and Oncology (AGIHO), Acute Myeloid Leukemia Cooperative Group (AML CG), Australian and New Zealand Mycoses Interest Group (ANZMIG), European Confederation of Medical Mycology (ECMM), European Hematology Association (EHA)—Study Working Group on Infections in Hematology, European Society for Blood and Marrow Transplantation (EBMT), FungiScope^®^ registry contributors, International Society for Human and Animal Mycology (ISHAM), Mycoses Study Group Education and Research Consortium (MSGERC), Epidemiological Surveillance of Infections in Hematological Diseases (SEIFEM), Austrian Society for Hematology and Medical Oncology (OeGHO), and Swiss Society of Hematology (SHG-SSH)**.** The participants were encouraged to respond for their medical center and spread the survey through their personal network.

All of the participants were asked to provide country and institution as an obligatory item, whereas additional personal data, affiliated scientific organizations, and practicing specialty were optional. The participants were encouraged to provide estimated annual numbers of patients that were treated at their hematology center with the following underlying conditions: AML (de novo/relapsed), acute lymphatic leukemia (ALL) (de novo/relapsed), and allogeneic HCT. The estimated rates of IPA in these patient groups were to be provided.

The timepoints and techniques of chest imaging for the respective underlying condition were assessed via a multiple-choice chessboard response form. Timepoints comprised at diagnosis, at staging, at admission, before each chemotherapy course, upon signs and symptoms of respiratory infection, at first fever, after 72–96 h of persistent fever, despite antibacterial treatment. The imaging techniques to be selected were X-ray, computed tomography (CT), or no imaging. All of the indicated timepoints of imaging may overlap. Baseline CT was not explicitly mentioned in order to obtain a non-suggestive assessment.

CT scan specificities, namely contrast-enhanced or non-contrast, low-dose or standard-dose, and evaluation of imaging through other specialties than radiology were assessed. Standard-of-care follow-up CT imaging for diagnosed IPA were to be selected on day 7, 14, 21, 28, or none.

BCT was defined as the performance of a CT scan upon admission or at diagnosis of the above-mentioned underlying conditions according to the two above-mentioned studies [14,15]. These two groups may overlap. For practicability of the survey, “HCT” was listed in diagnoses but also considered as medical history. Double-entries or invalid responses were not considered for statistical analysis. Initially, the findings of BCT were to be assessed. This approach was abandoned due to practical reasons, since even estimated determination of specific radiological findings for IPA is highly complex.

Free text options were provided wherever necessary.

Statistical analysis used SPSS software version 25 (IBM, Chicago, IL, USA). Participants’ data entries from categorical variables were summarized employing frequencies and percentages. Median and interquartile range (IQR) were used in the continuous variables. Categorical data were compared while using Chi^2^ test or Fisher’s exact test. A *p* value <0.05 was set as being statistically significant.

## 3. Results

Between July 1st and August 31st 2019, members of the above listed scientific societies entered data into the survey form.

**Figure 1:**
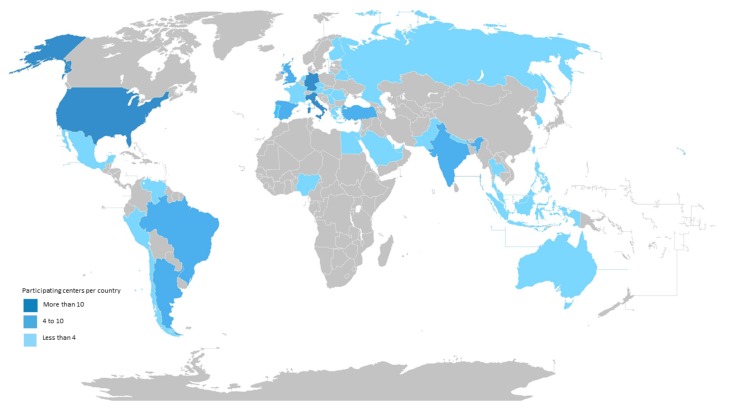
Geographic Distribution of Survey Participants.

Among 142 participants from 43 countries, 92 entered data for all of the questions. Seventy-nine (55.6%) participated in Europe and sixty-three (44.4%) participated outside Europe. Countries with highest participant numbers were Germany (*n* = 32; 22.5%), Italy, and the United States (*n* = 11; 7.7% each), followed by Brazil and Spain (*n* = 6; 4.2% each) (Table 1 and Figure 1).

Specialties involved were infectious diseases (*n* = 69; 48.6%), hematology (*n* = 68; 47.9%), microbiology (*n* = 15; 10.6%), intensive care (*n* = 8; 5.6%), oncology (*n* = 6; 4.2%), and pediatrics (*n* = 4; 2.8%) (Table 1).

The estimates of total overall annually treated patient numbers at participating hospitals (*n* = 101 responses) include 5505 AML (3736 de novo; 1769 relapsed), 2641 ALL (1817 de novo; 824 relapsed), and 5287 allogeneic HCT patients. The estimated median numbers of annually treated patients are 40 (IQR 18–70) for AML, 16 (IQR 7–35) for ALL, and 35 (IQR 2.5–75) for HCT (Table 2).

**Figure 2:**
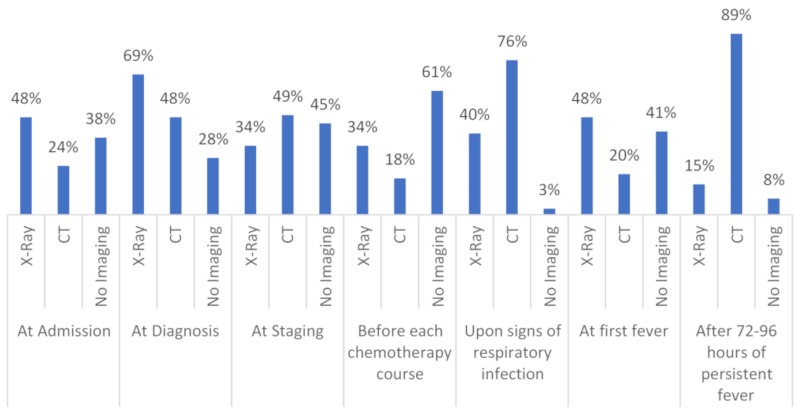
Shows the different timepoints and techniques of chest imaging in high-risk hematology patients in detail; *n* = 95. CT = computed tomography.

CT and/or X-ray across all patient groups is performed, as follows (*n* = 95 responses). At disease staging, X-ray is done in 34% (*n* = 32) and CT in 49% (*n* = 47), no imaging in 45% (*n* = 43). Before the beginning of chemotherapy administration, X-ray is performed in 34% (*n* = 32), CT in 18% (*n* = 17) and no imaging in 61% (*n* = 58). Upon initial signs or symptoms of pneumonia, X-ray is carried out in 40% (*n* = 38), CT in 76% (*n* = 72), and no imaging in 3% (*n* = 3). At first fever, X-ray is completed in 48% (*n* = 46) and CT in 20% (*n* = 19), while, at persistent fever, over 72 to 96 h 15% (*n* = 14) and 89% (*n* = 85) do X-ray or CT, respectively. In these two situations, 41% and 8% do not perform any imaging, respectively (Figure 2).

**Figure 3:**
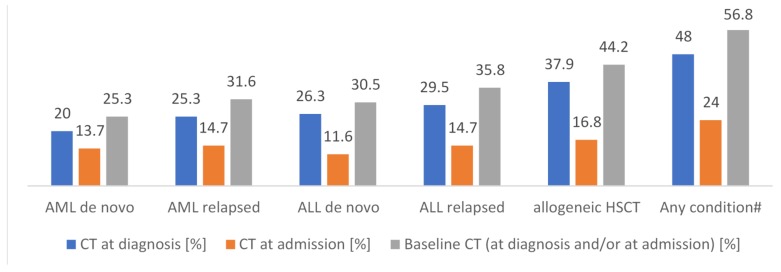
Underlying condition and performance of X-ray or CT at diagnosis or at admission, respectively—defined as Baseline CT; *n* = 95. # Numbers are super-additive. CT = computed tomography; AML = acute myeloid leukemia; ALL = acute lymphoblastic leukemia; HCT = hematopoietic cell transplantation.

At admission or at diagnosis of malignancy, X-ray is performed in 48% and 69% of participating centers, respectively. At the same timepoints, 24% and 48% of centers performed CT, respectively. As per our definition, this qualifies as BCT, which makes a proportion of 56.8% (*n* = 54) of centers performing BCT for any timepoint and condition (*n* = 95). Detailed BCT numbers at admission are 13.7% (*n* = 13) and 14.7% (*n* = 14) for de novo and relapsed AML, respectively, 11.6% (*n* = 11) and 14.7% (*n* = 14) for de novo and relapsed ALL, respectively, and 16.8% (*n* = 16) for HCT. The detailed BCT numbers at diagnosis are 20.0% (*n* = 19) and 25.3% (*n* = 24) for de novo and relapsed AML, respectively, 26.3% (*n* = 25) and 29.5% (*n* = 28) in de novo and relapsed ALL, respectively, and 37.9% (*n* = 36) for HCT. Overall, HCT is the underlying condition that is most frequently triggering BCT (44.2%; *n* = 42) and BCT is more frequently performed in relapsed than in *de novo* acute leukemia (Figure 3). The first implementation of BCT in a center was in 2010. Table S1 displays detailed timepoints, techniques, and numbers of chest imaging with respect to the underlying condition.

CT is contrast-enhanced in 37.6% (*n* = 35) and non-enhanced in 62.4% (*n* = 58). CT is low-dose in 37.6% (*n* = 35) and standard dose in 62.4% (*n* = 58) (total *n* = 93). The BCT scan specificities are 33.3% (*n* = 18) enhanced and 66.7% (*n* = 36) non-enhanced while 61.1% (*n* = 33) are standard-dose and 38.9% (*n* = 21) low-dose. In addition to radiology reports, the participants mostly read CT scans themselves. For BCT centers, this is the case in 87% (*n* = 47) (Table 3).

Overall, the median estimated rate of IPA is 8% (IQR 5 to 14%; *n* = 94). In non-BCT performing centers, it is only 7% (IQR 5-10%; *n* = 37), whereas it is 9% (5-15%; *n* = 52) in BCT centers (*p* = 0.69) (Table 4).

European centers perform BCT in 59.0% (*n* = 36), while non-European centers do so in 52.9% (*n* = 18). The reported estimated IPA rates do not differ between European and non-European centers (medians 10% vs. 8%; *p* = 0.25) (Table 4).

**Figure 4:**
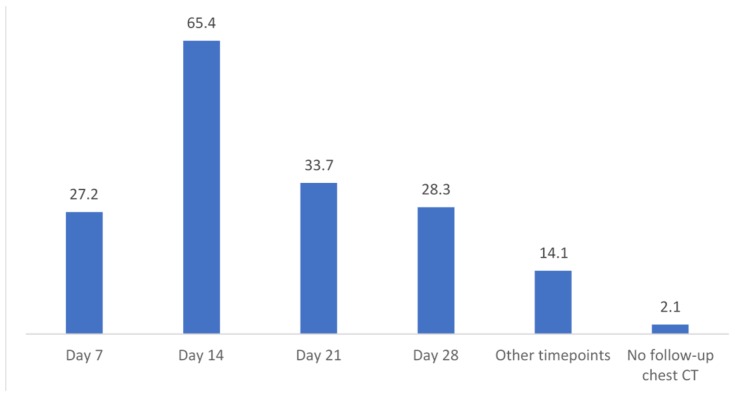
Timepoints and percentages of follow-up CT if IPA was diagnosed in a patient, *n* = 92 valid responses. CT = computed tomography; IPA = invasive pulmonary aspergillosis.

Follow-up CT imaging in the case of IPA diagnosis (*n* = 92) is performed in 97.8%. CT on day 7 after IPA diagnosis is performed in 27.2%, on day 14 in 65.4%, on day 21 in 33.7%, and on day 28 in 28.3% of centers. Other timepoints for follow-up CT were indicated in 14.1% (*n* = 13) (Figure 4).

## 4. Discussion

In this web-based survey study, 142 participants from 43 countries contributed to assessing current chest imaging use in high-risk hematology patients. BCT, defined as chest CT at diagnosis or at admission, is performed in 57% of centers to detect IPA early. The BCT rates in relapsed acute leukemia and in allogeneic HCT recipients are higher than in de novo acute leukemia [18]. As an expected finding, CT is mostly part of diagnostic-driven approaches, in particular in the case of persistent fever or suspected pneumonia, as recommended by guidelines [9,17].

Despite the acknowledged reduced diagnostic accuracy of plain radiography compared with CT for detection of IPA in neutropenic patients, it is still broadly used as a frontline investigation [19,20]. Chest X-ray was the preferred imaging procedure at baseline, although a negative X-ray calls for the more sensitive CT and a positive X-ray demands a standard or low-dose CT for its higher diagnostic specificity [19,20].

Low-dose CT detects and characterizes lung lesions in neutropenic patients early and with equal precision when compared to standard-dose CT [21,22]. Still, the use of standard-dose CT is more widespread than low-dose CT. When BCT is implemented, one needs to keep in mind that the effective radiation dose of modern techniques is less than that of a conventional standard dose CT [23]. Therefore, introducing low-dose BCT might provide acceptable radiation exposure and allow for early diagnosis of IPA with a lower fungal burden and improved patient outcome [24,25,26]. This point is particularly important for pediatric cancer patients as most will be long-term survivors. However, pediatric data for BCT is limited [27].

The median estimated IPA rates appeared to be higher with BCT in this survey, a finding that is in line with previous single center studies, but also underlying relatively small sample size and comprising the inherent bias of diagnosing more IPA episodes than without BCT [14,15]. The higher IPA rate suggest BCT as being likely beneficial for early diagnosis, also if asymptomatic. Early CT in hematological patients has been proposed to detect IPA at an early state of disease potentially improving patient outcome [28]. However, early findings on CT frequently do not match EORTC/MSG criteria and can delay diagnosis and subsequently deteriorate disease prognosis [29]. Therefore, in the revised EORTC/MSG radiological criteria, it was decided to include a broader range of radiological findings [13]. These were recently validated, revealing that nearly one-third of IPA patients presented with a consolidation pattern, but without typical nodules on the first CT [30].

Follow-up CT was more frequently indicated on day 14 than on day 7. Reflecting the course of infiltrate size, the aspergillosis EQUAL score recently suggested CT scans on days 7, 14, and then 21 or 28, but that is only practiced in 3% of the studied sites [11,31]. Some past studies did not favor baseline chest imaging due to low yield, although study design and population differed in their analyses [27,32]. To the knowledge of the authors, prospective clinical trials assessing baseline CT findings on patient outcome are lacking.

Our study has its limitations. First, 36% of respondents did not complete all of the questionnaire items. Second, only members of scientific bodies were invited to respond. Finally, frequencies were asked as estimates and may, thus, be imprecise. The widespread lack of in-house surveillance systems might make reported practices and fungal incidence imprecise. Our definition of BCT only comprises timepoints, but does not consider the heterogeneity of patient groups and their individual risk for IPA, such as heavily pre-treated patients that are admitted for HCT. The strengths of the study lie in a worldwide distribution of participants and an extensive analysis of chest imaging policies in high-risk hematology patients.

In conclusion, BCT in high-risk hematology patients has evolved into a standard of care in many centers worldwide, despite the absence of randomized controlled trials (RCTs) evaluating this procedure. However, chest X-ray, as the inferior technique, is still widely used. Low-dose CT techniques are apparently underused. Although much depends on local epidemiology, risk stratification of patients and local resources, centers considering the implementation BCT may find it useful for early diagnosis and the treatment of IPA in high-risk hematology patients.

## Figures and Tables

**Table 1:** Participant characteristics.

Country *n = 43*	*n*	[%]	Continent *n = 142*	*n*	[%]
Argentina	4	2.8	Europe	79	55.6
Australia	3	2.1	America	31	21.7
Austria	3	2.1	Asia	13	9.1
Belarus	1	0.7	Africa	15	10.5
Brazil	6	4.2	Oceania	4	2.8
Chile	1	0.7	**European vs. non-European countries**	***n***	**[%]**
Czech Republic	2	1.4	Non-European	63	44.4
Egypt	2	1.4	European	79	55.6
Finland	2	1.4	Medical specialty*	***n***	**[%]**
France	3	2.1	Infectious Diseases	69	48.6
Germany	32	22.5	Hematology	68	47.9
Hungary	1	0.7	Microbiology	15	10.6
India	4	2.8	Intensive Care	8	5.6
Indonesia	1	0.7	Oncology	6	4.2
Israel	2	1.4	Pediatrics	4	2.8
Italy	11	7.7	other	8	5.6
Lebanon	2	1.4			
Malaysia	3	2.1			
Mexico	1	0.7			
Nepal	1	0.7			
Netherlands	1	0.7			
Nigeria	1	0.7			
Pakistan	1	0.7			
Peru	3	2.1			
Philippines	1	0.7			
Portugal	1	0.7			
Qatar	1	0.7			
Romania	1	0.7			
Russia	2	1.4			
Saudi Arabia	1	0.7			
Serbia	2	1.4			
Singapore	1	0.7			
Republic of Korea	1	0.7			
Spain	6	4.2			
Switzerland	4	2.8			
Taiwan	2	1.4			
Thailand	1	0.7			
Turkey	5	3.5			
United Arab Emirates	1	0.7			
United Kingdom	5	3.5			
United States of America	11	7.7			
Venezuela	3	2.1			
Overall	142	100			

* numbers are super-additive.

**Table 2:** Patient numbers treated annually (at participating sites).

*n = 101*	AML Total	AML de novo	AML Relapsed	ALL Total	ALL de novo	ALL Relapsed	Allogeneic HCT *
Median (IQR)	40 (18–70)	25 (10–50)	10 (5–25)	16 (7–35)	10 (5–20)	5 (2–10)	35 (2.5–75)
(Min–Max)	0–377	0–300	0–150	0–206	0–150	0–60	0–400
Overall annually patient numbers	5505	3736	1769	2641	1817	824	5287

* this group may overlap with others. AML = acute myeloid leukemia; ALL = acute lymphoblastic leukemia; HCT = hematopoietic cell transplantation, IQR = interquartile range.

**Table 3:** Chest computed tomography (CT) specificities.

Chest CT Specificities			*n*	[%]
All centers n = 93 *	Chest CT	contrast-enhanced	35	37.6
		not contrast-enhanced	58	62.4
	Doses	Standard-dose CT	58	62.4
		Low-dose CT	35	37.6
	CT assessment ^#^	Yes	79	84.9
		No	14	15.1
Baseline CT centers only *n = 54*	Chest CT	contrast-enhanced	18	33.3
		not contrast-enhanced	36	66.7
	Doses	Standard-dose CT	33	61.1
		Low-dose CT	21	38.9
	CT assessment ^#^	Yes	47	87.0
		No	7	13.0

* total *n* of response to the respective question; ^#^ participants indicated if they evaluate chest imaging themselves despite not being radiologists. CT = computed tomography.

**Table 4:** Median estimated invasive pulmonary aspergillosis (IPA) rates.

*n*	Participating Centers	IPA Rate, Median (IQR)	*p*-Value
*n* = 94 *	Overall participating centers *n* = 94	8 (5–14)	-
*n* = 89 *	BCT centers *n* = 52	9 (5–15)	0.69
	non-BCT centers *n* = 37	7 (5–10)	
*n* = 94 *	European centers *n* = 58	10 (5–15)	0.25
	non-European centers *n* = 36	8 (5–14)	

*total *n* of valid responses to the respective questions. IPA = invasive pulmonary aspergillosis; BCT = baseline chest computed tomography.

## Supplementary Materials

The following are available online at https://www.mdpi.com/2309-608X/6/1/36/s1, Figure S1: title, Table S1: title, Video S1: title.

## References

[1] Steinbach W.J., Marr K.A., Anaissie E.J., Azie N., Quan S.P., Meier-Kriesche H.U., Apewokin S., Horn D.L. (2012). Clinical epidemiology of 960 patients with invasive aspergillosis from the PATH Alliance registry. J. Infect..

[2] Cornely O.A., Maertens J., Winston D.J., Perfect J., Ullmann A.J., Walsh T.J., Helfgott D., Holowiecki J., Stockelberg D., Goh Y.T. (2007). Posaconazole vs. fluconazole or itraconazole prophylaxis in patients with neutropenia. N. Engl. J. Med..

[3] Wald A., Leisenring W., Van Burik J.A., Bowden R.A. (1997). Epidemiology of *Aspergillus* infections in a large cohort of patients undergoing bone marrow transplantation. J Infect Dis.

[4] Koehler P., Hamprecht A., Bader O., Bekeredjian-Ding I., Buchheidt D., Doelken G., Elias J., Haase G., Hahn-Ast C., Karthaus M. (2017). Epidemiology of invasive aspergillosis and azole resistance in patients with acute leukaemia: The SEPIA Study. Int. J. Antimicrob. Agents.

[5] Pagano L., Caira M., Candoni A., Offidani M., Fianchi L., Martino B., Pastore D., Picardi M., Bonini A., Chierichini A. (2006). The epidemiology of fungal infections in patients with hematologic malignancies: The SEIFEM-2004 study. Haematologica.

[6] Pardo E., Lemiale V., Mokart D., Stoclin A., Moreau A.S., Kerhuel L., Calvet L., Valade S., De Jong A., Darmon M. (2019). Invasive pulmonary aspergillosis in critically ill patients with hematological malignancies. Intensive Care Med..

[7] Cattaneo C., Gramegna D., Malagola M., Pagani C., Borlenghi E., Cerqui E., Passi A., Sciume M., Bernardi S., Crippa C. (2019). Invasive pulmonary aspergillosis in acute leukemia: A still frequent condition with a negative impact on the overall treatment outcome. Leuk Lymphoma.

[8] Dib R.W., Hachem R.Y., Chaftari A.M., Ghaly F., Jiang Y., Raad I. (2018). Treating invasive aspergillosis in patients with hematologic malignancy: Diagnostic-driven approach versus empiric therapies. BMC Infect. Dis..

[9] Patterson T.F., Thompson G.R.I.I.I., Denning D.W., Fishman J.A., Hadley S., Herbrecht R., Kontoyiannis D.P., Marr K.A., Morrison V.A., Nguyen M.H. (2016). Practice Guidelines for the Diagnosis and Management of Aspergillosis: 2016 Update by the Infectious Diseases Society of America. Clin. Infect. Dis..

[10] Lewis R.E., Cahyame-Zuniga L., Leventakos K., Chamilos G., Ben-Ami R., Tamboli P., Tarrand J., Bodey G.P., Luna M., Kontoyiannis D.P. (2013). Epidemiology and sites of involvement of invasive fungal infections in patients with haematological malignancies: A 20-year autopsy study. Mycoses.

[11] Vehreschild J.J., Heussel C.P., Groll A.H., Vehreschild M., Silling G., Wurthwein G., Brecht M., Cornely O.A. (2017). Serial assessment of pulmonary lesion volume by computed tomography allows survival prediction in invasive pulmonary aspergillosis. Eur. Radiol..

[12] Ceesay M.M., Desai S.R., Berry L., Cleverley J., Kibbler C.C., Pomplun S., Nicholson A.G., Douiri A., Wade J., Smith M. (2015). A comprehensive diagnostic approach using galactomannan, targeted beta-d-glucan, baseline computerized tomography and biopsy yields a significant burden of invasive fungal disease in at risk haematology patients. Br. J. Haematol..

[13] Donnelly J.P., Chen S.C., Kauffman C.A., Steinbach W.J., Baddley J.W., Verweij P.E., Clancy C.J., Wingard J.R., Lockhart S.R., Groll A.H. (2019). Revision and Update of the Consensus Definitions of Invasive Fungal Disease From the European Organization for Research and Treatment of Cancer and the Mycoses Study Group Education and Research Consortium. Clin. Infect. Dis..

[14] Ceesay M.M., Desai S.R., Cleverley J., Berry L., Smith M., Wade J., Mufti G.J., Pagliuca A. (2018). Pre-symptomatic (Baseline) computed tomography predicts invasive pulmonary aspergillosis in high-risk adult haemato-oncology patients. Br. J. Haematol..

[15] Bitterman R., Hardak E., Raines M., Stern A., Zuckerman T., Ofran Y., Lavi N., Guralnik L., Frisch A., Nudelman O. (2019). Baseline Chest Computed Tomography for Early Diagnosis of Invasive Pulmonary Aspergillosis in Hemato-oncological Patients—A Prospective Cohort Study. Clin. Infect. Dis..

[16] Cornely O.A., Hoenigl M., Lass-Florl C., Chen S.C., Kontoyiannis D.P., Morrissey C.O., Thompson G.R. (2019). Defining breakthrough invasive fungal infection-Position paper of the mycoses study group education and research consortium and the European Confederation of Medical Mycology. Mycoses.

[17] Ullmann A.J., Aguado J.M., Arikan-Akdagli S., Denning D.W., Groll A.H., Lagrou K., Lass-Florl C., Lewis R.E., Munoz P., Verweij P.E. (2018). Diagnosis and management of *Aspergillus* diseases: Executive summary of the 2017 ESCMID-ECMM-ERS guideline. Clin. Microbiol. Infect..

[18] Kousha M., Tadi R., Soubani A.O. (2011). Pulmonary aspergillosis: A clinical review. Eur. Respir. Rev..

[19] Ramila E., Sureda A., Martino R., Santamaria A., Franquet T., Puzo C., Montesinos J., Perea G., Sierra J. (2000). Bronchoscopy guided by high-resolution computed tomography for the diagnosis of pulmonary infections in patients with hematologic malignancies and normal plain chest X-ray. Haematologica.

[20] Estacio O., Loh Z., Baker A., Chong G., Grigg A., Churilov L., Hawkes E.A. (2018). Limited utility of routine chest X-ray in initial evaluation of neutropenic fever in patients with haematological diseases undergoing chemotherapy. Intern. Med. J..

[21] Kubo T., Ohno Y., Takenaka D., Nishino M., Gautam S., Sugimura K., Kauczor H.U., Hatabu H. (2016). Standard-dose vs. low-dose CT protocols in the evaluation of localized lung lesions: Capability for lesion characterization-iLEAD study. Eur. J. Radiol. Open.

[22] Gerritsen M.G., Willemink M.J., Pompe E., Van der Bruggen T., Van Rhenen A., Lammers J.W., Wessels F., Sprengers R.W., De Jong P.A., Minnema M.C. (2017). Improving early diagnosis of pulmonary infections in patients with febrile neutropenia using low-dose chest computed tomography. PLoS ONE.

[23] Van der Bruggen-Bogaarts B.A.H.A., Broerse J.J., Lammers J.-W.J., Van Waes P.F.G.M., Geleijns J. (1995). Radiation Exposure in Standard and High-Resolution Chest CT Scans. CHEST.

[24] Cornely O.A., Maertens J., Bresnik M., Ebrahimi R., Dellow E., Herbrecht R., Donnelly J.P. (2011). Efficacy outcomes in a randomised trial of liposomal amphotericin B based on revised EORTC/MSG 2008 definitions of invasive mould disease. Mycoses.

[25] Greene R.E., Schlamm H.T., Oestmann J.W., Stark P., Durand C., Lortholary O., Wingard J.R., Herbrecht R., Ribaud P., Patterson T.F. (2007). Imaging findings in acute invasive pulmonary aspergillosis: Clinical significance of the halo sign. Clin. Infect. Dis..

[26] Caillot D., Casasnovas O., Bernard A., Couaillier J.F., Durand C., Cuisenier B., Solary E., Piard F., Petrella T., Bonnin A. (1997). Improved management of invasive pulmonary aspergillosis in neutropenic patients using early thoracic computed tomographic scan and surgery. J. Clin. Oncol..

[27] Kasow K.A., Krueger J., Srivastava D.K., Li C., Barfield R., Leung W., Horwitz E.M., Madden R., Woodard P., Hussain I. (2009). Clinical utility of computed tomography screening of chest, abdomen, and sinuses before hematopoietic stem cell transplantation: The St. Jude experience. Biol. Blood Marrow Transpl..

[28] Nucci M., Nouer S.A., Cappone D., Anaissie E. (2013). Early diagnosis of invasive pulmonary aspergillosis in hematologic patients: An opportunity to improve the outcome. Haematologica.

[29] Girmenia C., Guerrisi P., Frustaci A.M., Fama A., Finolezzi E., Perrone S., Gentile G., Collerone F., Brocchieri S., Guerrisi V. (2012). New category of probable invasive pulmonary aspergillosis in haematological patients. Clin. Microbiol. Infect..

[30] Herbrecht R., Guffroy B., Danion F., Venkatasamy A., Simand C., Ledoux M.-P. (2020). Validation by real-life data of the new radiological criteria of the revised and updated consensus definition for invasive fungal diseases. Clin. Infect. Dis..

[31] Cornely O.A., Koehler P., Arenz D., Mellinghoff S.C. (2018). EQUAL Aspergillosis Score 2018: An ECMM score derived from current guidelines to measure QUALity of the clinical management of invasive pulmonary aspergillosis. Mycoses.

[32] El Boghdadly Z., Oran B., Jiang Y., Rondon G., Champlin R., Kontoyiannis D.P. (2017). Pretransplant chest computed tomography screening in asymptomatic patients with leukemia and myelodysplastic syndrome. Bone Marrow Transpl..

