# Supplementary material for: Baseline Chest Computed Tomography as Standard of Care in High-Risk Hematology Patients
Source: J Fungi (Basel). 2020 Mar 13;6(1):36. doi: 10.3390/jof6010036 (PMC7151030; doi:10.3390/jof6010036)
Supplement: Supplementary file 1 [file jof-06-00036-s001.pdf]

Supplementary tables

Table S1. Exact timepoints, techniques and numbers of chest imaging with respect to the underlying condition

**Imaging Time**, n=95	**Imaging Technique**	**Condition**	**Number**	**[%]**
At diagnosis	X-Ray	AML de novo	62	65.3
		AML relapsed	55	57.9
		ALL de novo	58	61.1
		ALL relapsed	52	54.7
		allogeneic HSCT	43	45.3
	CT	AML de novo	19	20.0
		AML relapsed	24	25.3
		ALL de novo	25	26.3
		ALL relapsed	28	29.5
		allogeneic HSCT	36	37.9
	No Imaging	AML de novo	18	18.9
		AML relapsed	22	23.2
		ALL de novo	16	16.8
		ALL relapsed	20	21.1
		allogeneic HSCT	13	13.7
At staging	X-Ray	AML de novo	26	27.4
		AML relapsed	23	24.2
		ALL de novo	21	22.1
		ALL relapsed	20	21.1
		allogeneic HSCT	24	25.3
	CT	AML de novo	24	25.3
		AML relapsed	25	26.3
		ALL de novo	38	40.0
		ALL relapsed	38	40.0
		allogeneic HSCT	26	27.4
	No Imaging	AML de novo	40	42.1
		AML relapsed	38	40.0
		ALL de novo	31	32.6
		ALL relapsed	29	30.5
		allogeneic HSCT	29	30.5
At admission	X-Ray	AML de novo	41	43.2
		AML relapsed	37	38.9
		ALL de novo	38	40.0
		ALL relapsed	36	37.9
		allogeneic HSCT	36	37.9
	CT	AML de novo	13	13.7
		AML relapsed	14	14.7
		ALL de novo	11	11.6
		ALL relapsed	14	14.7
		allogeneic HSCT	16	16.8

	No Imaging	AML de novo	31	32.6
		AML relapsed	31	32.6
		ALL de novo	33	34.7
		ALL relapsed	33	34.7
		allogeneic HSCT	23	24.2
Before every chemotherapy treatment cycle	X-Ray	AML de novo	28	29.5
		AML relapsed	26	27.4
		ALL de novo	21	22.1
		ALL relapsed	23	24.2
		allogeneic HSCT	22	23.2
	CT	AML de novo	8	8.4
		AML relapsed	7	7.4
		ALL de novo	6	6.3
		ALL relapsed	7	7.4
		allogeneic HSCT	13	13.7
	No Imaging	AML de novo	50	52.6
		AML relapsed	49	51.6
		ALL de novo	55	57.9
		ALL relapsed	52	54.7
		allogeneic HSCT	39	41.1
Upon signs or symptoms of respiratory infection	X-Ray	AML de novo	35	36.8
		AML relapsed	35	36.8
		ALL de novo	36	37.9
		ALL relapsed	35	36.8
		allogeneic HSCT	32	33.7
	CT	AML de novo	68	71.6
		AML relapsed	69	72.6
		ALL de novo	64	67.4
		ALL relapsed	69	72.6
		allogeneic HSCT	63	66.3
	No Imaging	AML de novo	0	0.0
		AML relapsed	0	0.0
		ALL de novo	1	1.1
		ALL relapsed	0	0.0
		allogeneic HSCT	2	2.1
First fever	X-Ray	AML de novo	41	43.2
		AML relapsed	40	42.1
		ALL de novo	40	42.1
		ALL relapsed	41	43.2
		allogeneic HSCT	37	38.9
	CT	AML de novo	11	11.6
		AML relapsed	12	12.6
		ALL de novo	10	10.5
		ALL relapsed	11	11.6
		allogeneic HSCT	12	12.6
	No Imaging	AML de novo	36	37.9
		AML relapsed	33	34.7
		ALL de novo	37	38.9

		ALL relapsed	34	35.8
		allogeneic HSCT	30	31.6
72-96h persistent fever	X-Ray	AML de novo	11	11.6
		AML relapsed	11	11.6
		ALL de novo	12	12.6
		ALL relapsed	10	10.5
		allogeneic HSCT	10	10.5
	CT	AML de novo	82	86.3
		AML relapsed	82	86.3
		ALL de novo	78	82.1
		ALL relapsed	82	86.3
		allogeneic HSCT	74	77.9
	No Imaging	AML de novo	4	4.2
		AML relapsed	4	4.2
		ALL de novo	5	5.3
		ALL relapsed	5	5.3
		allogeneic HSCT	5	5.3

CT=Computed Tomography; AML=Acute myelogenous leukemia; ALL=acute lymphoblastic leukemia; HSCT=hematopoietic stem cell transplantation.
